# Symbiotic Associations: Key Factors That Determine Physiology and Lipid Accumulation in Oleaginous Microorganisms

**DOI:** 10.3389/fmicb.2020.555312

**Published:** 2020-12-16

**Authors:** Deepi Deka, Shashanka Sonowal, Channakeshavaiah Chikkaputtaiah, Natarajan Velmurugan

**Affiliations:** ^1^Biological Sciences Division, Branch Laboratory-Itanagar, CSIR-North East Institute of Science and Technology, Naharlagun, India; ^2^Academy of Scientific and Innovative Research (AcSIR), CSIR-NEIST, Jorhat, India; ^3^Biological Sciences and Technology Division, CSIR-North East Institute of Science and Technology, Jorhat, India

**Keywords:** oleaginous microorganisms, symbiotic associations, physiology, evolutionary relationships, artificial symbiosis

## Abstract

Symbiosis naturally provides an opportunity for microorganisms to live together by mutual or one-way benefit. In symbiotic relationships, the microorganisms usually overcome the limitations of being free-living. Understanding the symbiotic relationships of oleaginous microorganisms provides potential route for the sustainable production of microbial-based alternative fuels. So far, several studies have been conducted in oleaginous microorganisms for the production of alternative fuels. However, some oleaginous microorganisms require high quantity of nutrients for their growth, and high level of energy and chemicals for harvest and separation of lipid bodies. Symbiotic associations can successfully be applied to address these issues. Of symbiotic associations, lichens and selective species of oleaginous endosymbiotic mucoromycotina have received substantial interest as better models to study the evolutionary relationships as well as single-cell oil production. Construction of artificial lichen system composed of cyanobacteria and oleaginous yeast has been achieved for sustainable production of lipids with minimum energy demand. Recently, endosymbiotic mucoromycotina species have been recognized as potential sources for biofuels. Studies found that endohyphal bacterium influences lipid profiling in endosymbiotic mucoromycotina species. Studies on the genetic factors related to oleaginous characteristics of endosymbiotic mucoromycotina species are scarce. In this regard, this review summarizes the different forms of symbiotic associations of oleaginous microorganisms and how symbiotic relationships are impacting the lipid formation in microorganisms. Further, the review also highlights the importance of evolutionary relationships and benefits of co-culturing (artificial symbiosis) approaches for sustainable production of biofuels.

## Introduction

The evolution and beauty of symbiotic associations can simply be explained by studying the lichens. Lichens, lower plants composed of photosynthetic microorganisms (microalgae or cyanobacteria) and filamentous microorganisms (fungi), have been investigated intensely as a robust model system to understand symbiotic associations between different groups of microbes (Bonfante, 2019). Interestingly, photosynthetic microorganisms that emerge as lichens were well known for the accumulation of intracellular fatty acids. The unique ability of fatty acids accumulating microorganisms to establish a stable symbiotic association with filamentous fungi depends on several factors including mutual nutrient exchange (Santos and Reis, 2014). The physical and metabolic interactions between these microorganisms establish long-term and stable mutual symbiotic associations (Du et al., 2019). These features of symbiotic association between oleaginous microbes and associated microbes can be exploited heavily in biotechnology to develop bioprocessing and metabolic engineering of oleaginous microbes for various biotechnological applications.

In general, two phases are involved in accumulation of excessive amount of intracellular fatty acids in oleaginous microorganisms (Davies and Holdsworth, 1992). The oleaginous microbe consumes available nitrogen for cell growth and proliferation in the first stage and further converts available carbon into intracellular fatty acids mainly under nitrogen limitation condition (Davies and Holdsworth, 1992; Sonowal et al., 2019). Symbiotic association helps to overcome few barriers associated with oleaginous microbes hence reducing cost associated with production of microbial biofuels. Mainly, symbiotic association between oleaginous microalgae (or bacteria) and filamentous fungi allows flocculation of oleaginous microbes, hence reducing cost associated with harvesting process (Du et al., 2018; Arora et al., 2019).

Several reports have studied the establishment of symbiotic associations between oleaginous microbes and their associate partners in natural ecosystems, as well as in artificial symbiotic (co-cultivation) systems (Lastovetsky et al., 2016; Li et al., 2017; Du et al., 2019). Recently, we conducted a comparative phenotypic and genomic characterization of sugar excreting endosymbiont *Micractinium* sp. of a unicellular ciliate *Paramecium bursaria* (Arriola et al., 2018). The unique ability of *Micractinium* to establish a stable symbiotic association with *P. bursaria* depends on transfer of certain carbon compounds to host *Paramecium* for nutrients. Endosymbiotic *Micractinium* species have been reported to release the maximum level of sugars, especially maltose, into extracellular environment, at specific pH value of 5.7. This excretion scenario enables another critical advantage of the creation of an artificial microalgal-bacterial symbiosis to drive the sustainable production of various bioproducts. However, replicating microbial symbiotic systems in laboratory conditions as existing in natural ecosystems is highly difficult because of hurdles in systematic cultivations and experimental manipulations. Therefore, as of now, little is known about the molecular mechanisms responsible for establishment and maintenance of these symbiotic associations in natural ecosystems (Lastovetsky et al., 2016). In this context, the first part of this mini-review highlights biodiversity, physiology, and evolutionary relationship of oleaginous microbe with respect to symbiotic associations. The advantage of co-cultivation strategies for the development of industrial strains of oleaginous microorganisms is discussed in the second part.

## Biodiversity and Distribution of Oleaginous Symbiotic Microorganisms

Oleaginous microorganisms are considered as single-cell oil producers capable of producing more than 20–25% lipid in their dry cell weight; exceptionally, it can accumulate up to 60% in some cases (Table 1). As evaluated in few recent reports, with respect to symbiotic associations, the biodiversity of oleaginous microbes is widespread in nature (Thatoi et al., 2013; El-haj et al., 2015; Jiru et al., 2016; Martinez et al., 2016). This widespread diversity range allows their existence in the environment in two fundamental symbiotic forms, ectosymbiosis and endosymbiosis.

**TABLE 1:** List of some of oleaginous symbiotic microorganisms with intracellular lipid accumulation ≥40% and their substrate utilizations.

Oleaginous microorganisms	Habitats	Substrate utilized as carbon source	Lipid content (%)	References
**Ectosymbiotic microorganisms fungi**				
*Cryptococcus luteolus* InaCC Y-265	*Piper betle* and *P. nigrum*	Phenolic compounds	42.80	Kanti et al. (2013)
*Candida orthopsilosis* InaCC Y-302			42.78	
*C. oleophila* InaCC Y-306			42.80	
*Cystofilobasidium infirmominiatum* PL1	Lemon	Crude glycerol	40	Pereyra et al. (2014)
*Cryptococcus curvatus* PY39	Flower	–	46.51	Jiru et al. (2016)
*Mucor circinelloides* Q531	Mulberry branches	Cellulose, hemicellulose and lignin	42.43	Qiao et al. (2018)
**Fungal-like organisms**				
*Aurantiochytrium* sp. JMVL8	Leaf litter (mangrove)	Tributyrin, urea	60	Jaseera et al. (2018)
**Yeast**				
*Yarrowia lipolytica* NC-A	Wheat	Various forms of oil wastes	45.49	Bialy et al. (2011)
*Y. lipolytica* NC-D	Lettuce		57.89	
*Y. lipolytica* NC-I	Guava		61.64	
*Rhodotorula glutinis*	*Rose centifolia*	Xylose	36.60	Dai et al. (2007)
*Candida* sp. LEB-M3	Plant sources from Pantanal	Glycerol	56.58	Duarte et al. (2013)
**Bacteria**				
*Pseudomonas* sp. RRL-28	*Heteronema erecta*	Glucose	42.70	Patnayak and Sree (2005)
**Endosymbiotic microorganisms fungi**				
*Colletotrichum* sp. DM06	*Ocimum sanctum*	Lignocellulose	49.10	Dey et al. (2011)
*Alternaria* sp. DM09	*Brassica juncea*		58.10	
*Fusarium* sp. Soy1 A-3	Soyabean	Lignocellulose	44.95	Yang et al. (2014)
*Fusarium* sp. Soy1 A-4			40.61	
*Fusarium* sp. Soy1 A-11			42.82	
*Fusarium* sp. Soy1 A-17			47.56	
*Fusarium* sp. Soy1-32			43.17	
*Fusarium* sp. ML-GEN.1	*Strobilanthes cusia*	Molasses	59	Xie et al. (2013)
**Bacteria**				
*Bacillus subtilis* HB1310	Thin-shelled walnut	Lignocellulosic hydrolysate	40	Zhang et al. (2014)

## Oleaginous Ectosymbiotic Microorganisms

Ectosymbiotic microorganisms live on the surface of the host and maintain stable relationships with the host. Majority of reported oleaginous ectosymbiotic microorganisms maintain their relationships with hosts in the form of commensalism in which oleaginous microbes get benefited from the host. And, specific ectosymbiotic microorganisms were reported for mutualistic interactions (Bago et al., 2002). In the mutualistic alliance between arbuscular mycorrhizal (AM) fungi and plants, the fungus utilizes plant hexose as carbon source and stored carbons predominantly as triacylglycerol (TAG) (Bago et al., 2002). To maintain the AM symbiosis, the stored TAGs achieved the bidirectional movement between the intraradical mycelium to extraradical mycelium. Further, a substantial recirculation of TAGs throughout the fungus was also observed in order to maintain the AM symbiosis (Bago et al., 2002). Although AM fungi were classified as oleaginous as they can accumulate over 25% of intracellular lipids, the accumulation of lipids and utilization pattern in AM symbiotic fungi appeared to be different from other oleaginous fungi. In AM symbiosis, fungus utilizes plant carbons to synthesis storage lipids in intraradical mycelium and distributed throughout fungal mycelium. By this strategy, translocation of TAGs ensures availability of carbon throughout the mycelium (Bago et al., 2002). Specific strains of oleaginous microbes were found to be associated with a wide range of habitats including flower, rainforest trees, lawns, mangrove leaves, dairy products, sponges, and zooplankton (Dai et al., 2007; Li et al., 2012; Unagul et al., 2017). In general, habitats with high C:N ratio were reported to be better for microbial lipid accumulation (Li et al., 2012). Among oleaginous ectosymbiotic fungi, thraustochytrids (fungal-like microorganisms) are an important group of oleaginous microbes that establish very strong ectosymbiotic association with their hosts, mainly decaying mangrove leaves (Unagul et al., 2017). In addition, this group is reported for their associations with a wide range of invertebrates including sponges, hydroids, biovalves, and zooplankton (Marine Alliance for Science and Technology for Scotland [MASTS], 2020). Thraustochytrids have received much attention because the group potentially accumulates fatty acids up to 60%, especially highly prized fatty acids (Table 1). Of different thraustochytrids species, *Schizochytrium*, *Thraustochytrium*, *Aurantiochytrium*, and *Ulkenia* have been reported to accumulate a high level of polyunsaturated fatty acids (Chang et al., 2014). Fatty acid composition of thraustochytrids were mainly dominated by PA-C16, EPA-C20:5, AA-C20:4, DPA-C22:5, and DHAC22:6 (Kobayashi et al., 2011). Apart from thraustochytrids, the manglicolous oleaginous fungi and yeast have been reported to utilize carboxymethyl cellulose, dextrose, lignocelluloses, and xylose as sole carbon sources and produce high level of intracellular fatty acids (Khot et al., 2012; Thatoi et al., 2013). The lipid bodies of mangrove inhabited oleaginous fungi and yeast were mainly composted of saturated and monounsaturated fatty acids including PA-C16, SA-C18:0, and OA-C18:1 (Khot et al., 2012; Thatoi et al., 2013). Several oleaginous fungal strains were isolated from lawns of Tibetan Plateau, and all lawn inhabiting fungal strains utilized xylose and carboxymethyl cellulose as sole carbon sources (Li et al., 2012). Oleaginous yeast strain *Rhodotorula glutinis* isolated from leaves of *Rose centifolia* accumulated intracellular lipid up to 36.6% while utilizing xylose as a sole carbon source (Dai et al., 2007). Another study by Duarte et al. (2013) isolated oleaginous yeast strain of *Candida* sp. from a plant of Pantanal Tropical Wetland Area of Brazil, and this strain accumulated more than 56.68% of intracellular lipid while utilizing raw glycerol. In the view of above studies, isolation of oleaginous ectosymbiotic microbial strain from materials composed of lignocelluloses would be of great significance as these isolates can potentially excrete various hydrolytic enzymes with better capabilities. Oleaginous ectosymbiotic fungal, yeast, and bacterial species were also isolated from various dairy products including artisanal cheese, kefir, etc (Mattanna et al., 2014; Gientka et al., 2017).

## Oleaginous Endosymbiotic Microorganisms

Endosymbiosis is defined as the tendency of living inside the host body or cells of another organism in a mutualistic relationship. Endophytes are considered to be of great resources for the isolation of novel secondary metabolites and offering several potential compounds for various sectors including agriculture, medical, and oil industries (Strobel and Daisy, 2003). Oleaginous plant species can be the best hosts for the isolation of oleaginous microbial species, which can potentially accumulate high level of lipids (Peng and Chen, 2007). It has been reported that due to horizontal gene transfer between the hosts and their endosymbiotic microbes, the endosymbionts can produce the same metabolite as their hosts (Stadler and Schulz, 2009). Therefore, the endosymbionts originally derived from oleaginous plants can have higher capability to accumulate high level of lipids (Yang et al., 2014). For example, several lipid-accumulating endophytic fungal strains were isolated from oil-rich seed crops such as soybeans, sunflowers, and canola (Peng and Chen, 2007; Dey et al., 2011; Venkatesagowda et al., 2012). Myco-diesel composed of volatile hydrocarbons has been identified in oleaginous fungal strain *Gliocladium roseum*, originally an endophyte of ulmo plant *Eucryphia cordifolia* (Strobel et al., 2008). It is very interesting to look into the ecophysiological aspects in this alliance between *G. roseum* and *E. cordifolia*; *G. roseum* requires little free oxygen to grow better and produces good quantity of volatile hydrocarbons. This may be due to the fact that *G. roseum* synthesized volatile hydrocarbons as its adaptation to limited free oxygen environment inside the plant *E. cordifolia*. On the other hand, majority of plant-associated fungal endophytes were able to degrade cellulose and utilize digested components for the production of hydrocarbons. This hydrolytic processing of cellulose degradation requires high level of oxygen and difficult to achieve in little free-oxygen environment. Therefore, it was proposed that endophytic *G. roseum* produces volatile hydrocarbons in a different biosynthetic pathway independent of cellulose degradation and utilization pathways (Stadler and Schulz, 2009). Therefore, Strobel et al. (2008) suggested increasing the volatile hydrocarbon production in *G. roseum* by genetic manipulation and innovative fermentation technology. In addition to this fact, in industrial point of view, the ability to secrete important hydrolytic enzymes is an additional advantage of oleaginous endophytes as they may have the ability to decompose cheap plant raw materials such as straw while producing microbial oils (Osono and Takeda, 1999; Suto et al., 2002; Peng and Chen, 2007; Dey et al., 2011; Yang et al., 2014). In this context, several oleaginous endophytic fungal species (*Microsphaeropsis*, *Phomopsis*, *Cephalosporium*, *Colletotrichum*, *Alternaria*, *Sclerocystis*, *Nigrospora*, and *Cunninghamella* sp.) were isolated from various oleaginous plant species (*Keteleeria* sp., *Taxus chinensis*, *Sabina chinensis*, *Ocimum sanctum*, *Brassica juncea*, and *Salicornia bigelovii*) (Peng and Chen, 2007; Dey et al., 2011; Zhao et al., 2014).

## Physiology and Evolutionary Relationship of Oleaginous Symbiotic Microorganisms With Respect to Intrinsic Lipid Accumulation

**FIGURE 1:**
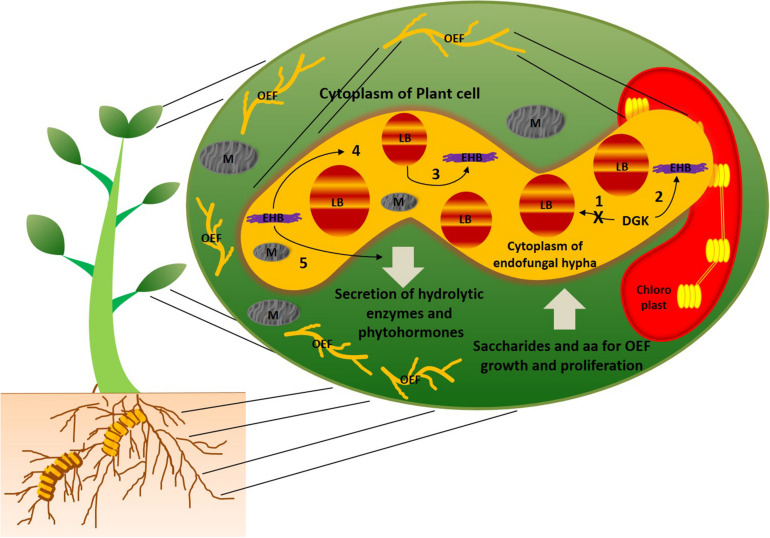
Schematic representation of symbiotic associations between plant, oleaginous endophytic fungal species of mucoromycotina (OEF), and endohyphal bacteria (EHB). Plant cytoplasm harbors OEF, and OEF hyphal cytoplasm harbors EHB. Majority of fungal species of mucoromycotina reported to possess oleaginous characteristics (Takeda et al., 2014). EHB maintains facultative relationships with OEF and reported to influence the physiology, secondary metabolite production, and intracellular lipid profiling in OEF (Hoffman et al., 2013; Lastovetsky et al., 2016). DGK found to be key enzymes that regulate lipid metabolism in OEF under the influence of EHB (Lastovetsky et al., 2016). The possible metabolic scenarios of this trio combination are as follows: (1) when DGK activity was inhibited, enhanced accumulation of intracellular fatty acids was found in OEF and symbiotic association between OEF and EHB shifted to be antagonistic; (2) when DGK activity was increased, accumulation of intracellular fatty acids found to suppressed in OEF and symbiotic association between OEF and EHB shifted to be mutualistic (Lastovetsky et al., 2016); (3) cytoplasmic fatty acids of OEF were utilized by EHB as sole carbon sources for their growth; (4) EHB was found to be responsible for pathogenicity of OEF via production of phytotoxins, and OEF lost its virulence in absence of EHB (Partida-Martinez and Hertweck, 2005); (5) EHB induces phytohormone production in OEF (Hoffman et al., 2013). In symbiotic association between OEF and host plants, OEF secretes hydrolytic enzymes and phytohormones into the cytoplasm of the host while obtaining saccharides and amino acids for their growth and proliferation. AA, amino acids; LB, lipid bodies; M, mitochondria.

In general, bacteria and fungi are known as natural antagonists to each other. Over the period of evolution, they are able to establish mutual symbiotic associations between them with a drastic change in lipid metabolism in at least one of the counterparts. Studies on mutual associations between oleaginous microorganisms can help improve their role in oil production. However, little information is available on the molecular mechanisms behind symbiotic associations of oleaginous symbiotic microorganisms. As we mentioned earlier, this may be due to practical hurdles on establishing these symbiotic associations in the laboratory conditions. Selective fungal species of mucoromycotina were recognized as oleaginous species (Takeda et al., 2014). *Umbelopsis isabellina* and *Cunninghamella echinulata* of mucoromycotina family were reported to accumulate more than 40% lipids (Fakas et al., 2009). It is interesting to observe that majority of oleaginous mucoromycotina family species were endophytes of plants; further, several bacteria were found to live within hyphae of these fungal endophytes, and they were termed as endohyphal bacteria (Hoffman and Arnold, 2010; Hoffman et al., 2013). This trio combination of plant-oleaginous endophytic fungi-endohyphal bacteria creates phylogenetically diverse array of symbiosis (Hoffman and Arnold, 2010). Figure 1 illustrates the symbiotic associations between this trio combination. The endohyphal bacteria were assumed to maintain facultative relationships with the oleaginous endophytic fungi (Hoffman et al., 2013). However, endohyphal bacterial species were reported to influence physiology (or phenotype) of oleaginous endophytic fungi, as well as symbiotic associations between oleaginous endophytes and the hosts (Cannone et al., 2002; Bonfante and Anca, 2009; Shaffer et al., 2016). It is well known that fungi and insects were sharing common metabolic pathways, such as chitin biosynthesis, and this suggests a possibility of an evolutionary transition of genetic material between the plant–endophytic fungi–endohyphal bacteria (Bonfante and Desiro, 2017). A study by Lastovetsky et al. (2016) reported the molecular mechanisms responsible for mutualistic relationships between oleaginous fungus *Rhizopus microsporus* and endohyphal bacteria *Burkholderia*. The study revealed that *R. microsporus* and *Burkholderia* symbiotic association altered the lipid profiles in the endophytic fungus. Diacylglycerol kinase (DGK) enzymes were found to regulate lipid metabolism in the oleaginous endophytic fungus, *R. microsporus*, when mutual symbiosis was established. Most importantly, when DGK activity was reduced, the fatty acids contents were increased in *R. microsporus*, and symbiotic interactions shifted into antagonism. DGK enzymes involved in these symbiotic associations were separately categorized from already reported DGKs, and this may because of an ancient horizontal gene transfer event. This mutualistic evolution provides more insights into genetics and biochemistry of lipid metabolism in this oleaginous endophytic fungus. Intracellular fatty acids of *R. microsporus* can potentially be utilized by endohyphal *Burkholderia* as a sole carbon source (Lackner et al., 2011). In addition, endohyphal bacteria also acquire carbon, nitrogen, and phosphorus from their endophytic fungal hosts (Salvioli et al., 2010; Ghignone et al., 2012). Apart from this, endohyphal *Burkholderia* were also found to be responsible for the pathogenicity of *R. microsporus* via production of phytotoxin (Partida-Martinez and Hertweck, 2005). The oleaginous fungal endophyte was found to be non-virulent in the absence of endohyphal bacteria. Genome sequencing of soil-inhabiting fungus *Mortierella elongata* and its endohyphal bacterium *Mycoavidus cysteinexigens* revealed that the fungal host metabolism was highly influenced by its endohyphal bacterium (Uehling et al., 2017). On the other hand, *Mortierella alpina*, an oleaginous endophytic fungus of the costliest spice plant, *Crocus sativus*, enhanced the production of apocarotenoids in *C. sativus*, as well as tolerance of *C. sativus* to corm rot disease by releasing arachidonic acid, and induced jasmonic acid production in *C. sativus* (Wani et al., 2017). The study revealed that *M. alpina* regulates and influences secondary metabolites pathways and production in the host.

Naturally, in the majority of algal–fungal symbiotic associations, algal cells were hypothesized to be on the surface of the fungal cells. Isotopic studies on the synthetic symbiotic associations between oleaginous alga *Nannochloropsis oceanica* and fungus *M. elongata* documented the association of active algal cells internalized within the fungal hyphae (Du et al., 2019). The unique ability of *N. oceanica* to establish a stable symbiotic association with *M. elongata* depends on several factors including transfer of carbon and nitrogen between the fungi and algae. Overall, comparative studies between the oleaginous symbiotic microorganisms and their hosts can provide useful information regarding the genetic factors related to oleaginous characteristics of the microorganisms. This can further help us to develop these species as potential strains for industrial applications.

## Co-Culturing Approaches: The Emergence of Artificial Symbiosis and Its Benefits

Despite the advantageous characteristics of oleaginous microorganisms, bioprocessing techniques, especially efficient production in large scale and harvesting, are still improved to make them competitive enough to compensate the global requirements for fossil fuels (Dias et al., 2019). Most importantly, harvesting consumes up to 50% of the cost of the biofuel production as it requires energy and chemicals (Wrede et al., 2014). Bioflocculation can be an attractive simple method because it significantly reduces energy and chemicals, hence maximizing the harvesting efficiency (Du et al., 2018). Majority of co-culturing (or artificial symbiosis) works have been reported with microalgae. Artificial symbiosis of oleaginous microalgae with other microalgae, bacteria, yeast, and filamentous fungi has gained interest because of enhanced lipid production obtained as well as highly efficient bioflocculation (Magdouli et al., 2016). In addition, co-culturing also offers several other applications including production of extracelluar polymeric substances (EPS), poly hydroxy alkanoates (PHA), organic acids, ethanol, biohydrogen, and electricity (Magdouli et al., 2016). A review by Magdouli et al. (2016) briefly described about the advantages and disadvantages of co-culturing of oil producing microbes. Here, we describe some of the key achievements in co-culturing of oleaginous microbes. Co-culturing of oleaginous yeast *Trichosporonoides spathulata* and *Chlorella vulgaris* yields 47% of lipid content (Kitcha and Cheirsilp, 2014). While co-culturing microalgae and yeasts species, yeast usually grows faster, and provided microalgae act as oxygen generator for yeast; meanwhile, yeast provided CO_2_ to microalga, further converting available nitrite to ammonium, and both species accumulate intracellular lipids (Cheirsilp et al., 2011; Papone et al., 2012; Naidoo et al., 2019). A study by Du et al. (2018) developed a bioflocculation method using combination of oleaginous microalgae *N. oceanica* and filamentous fungus *M. elongata* and synergistically increased fatty acid content. The study supports that synergistic interaction between oleaginous microalgae and filamentous fungi reduces costs related to nutrient supply, harvesting, and lipid extraction. In another study, the flocculation efficiency of filamentous fungus *Aspergillus fumigatus* was evaluated against 11 microalgal strains including photoautotrophs and heterotrophs, and this co-culturing resulted in enhanced biomass and lipids (Wrede et al., 2014). A combination of mixed cultures of *A. fumigatus* and oleaginous cyanobacterial species *Synechocystis* was found to be grown in animal husbandry wastewaters (Miranda et al., 2015). While co-culturing oleaginous microalgae with filamentous fungi or yeast, we can potentially reduce cost related to nutrients supply. As majority of filamentous fungi and engineered yeast strains can secrete hydrolytic enzymes, they can degrade the cheap ligninocellulosic materials or other agroindustrial wastes; further, the oleaginous microalgae can utilize the simple sugars out of degraded compounds for their growth and lipid production. In addition, fungal hydrolytic enzymes can convert selective microalgal strains into the cell wall–free protoplast, hence reducing the requirements of organic solvents used for lipid extraction (Wrede et al., 2014; Muradov et al., 2015).

Most recently, a study by Li et al. (2017) established an artificial lichen system composed of photosynthetic cyanobacteria *Synechococcus elongatus* and oleaginous yeast *R. glutinis*. This artificial lichen formation resulted in strong symbiotic and synergistic interactions for sustainable production of lipids from sunlight and CO_2_. A major benefit of photobiont and heterotroph combination is that the heterotroph can efficiently convert the available carbon into metabolites, and these metabolites can act as precursors for lipid accumulation (Li et al., 2017).

## Conclusion and Prospective

Symbiotic associations of oleaginous microorganisms are recognized as potential and sustainable biomass sources for lipid production and play a vital role in the development of bioprocessing of oleaginous microorganisms. Exploring the molecular mechanisms responsible for establishment of symbiotic associations, especially endosymbiotic, has tremendous advantage for the improvement of strain at an industrial scale. For example, finding the key factors that trigger establishment of symbiotic associations and transportation of beneficial products over symbiotic partners will help us to engineer candidate strains for commercial applications. Establishment of artificial symbiosis provides a great opportunity for systematic cultivation of oleaginous microorganisms at controlled conditions. However, few considerable challenges still remain including selection of suitable strain, controlling the growth and dominance of one species over symbiotic partner, and engineering partners to grow on waste biomass. Increasing the availability of complete genome sequences of oleaginous symbiotic microorganisms can make it easier for these functional studies and intensive engineering. Finally, the improved designs of artificial symbiosis in oleaginous microorganisms can be used for various biotechnological applications.

## Author Contributions

NV formulated the manuscript. DD, SS, CC, and NV performed the literature screening and wrote the article. SS and NV designed the figure. All authors contributed to the article and approved the submitted version.

## Conflict of Interest

The authors declare that the research was conducted in the absence of any commercial or financial relationships that could be construed as a potential conflict of interest.
